# *OVI*-Guided Tuning of Oxygen Vacancies in Fly Ash Denitrification Catalysts

**DOI:** 10.3390/ma18225229

**Published:** 2025-11-19

**Authors:** Zhanfeng Qi, Shuyang Wang, Xiuli Guo, Guancheng Li, Jingliang Li

**Affiliations:** College of Mechanical Engineering, Dalian University, Dalian 116622, China; qizhanfeng@dlu.edu.cn (Z.Q.); 18249461519@163.com (S.W.); lgc991116@outlook.com (G.L.); 13050373234@163.com (J.L.)

**Keywords:** oxygen vacancy, denitrification catalysis, defect modulation, defect engineering, Oxygen Vacancy Index (*OVI*), fly ash reuse

## Abstract

Denitrification catalysts are essential for reducing NO_x_ emissions from combustion and protecting air quality. This study introduces and validates an Oxygen Vacancy Index (*OVI*) that quantifies redox-active vacancies and guides defect engineering in fly ash-derived catalysts. We apply a simple cascade strategy to tune defect types; procedural details are reported in Methods rather than in the abstract. The optimized catalyst shows an over-twofold increase in *OVI* compared with raw fly ash. Surface-related changes and point/line defects contribute comparably (≈one-third each) to the explained variance. *OVI* positively correlates with NO conversion, and the optimized material delivers high NO conversion at 400 °C. These results establish a quantitative process–structure–performance link. Looking ahead, the *OVI* framework can guide defect design in other waste-derived catalysts and support scale-up to monolith coatings and pilot-scale units. This *OVI*-guided route provides a simple, low-cost path to robust fly ash catalysts for industrial NO_x_ control.

## 1. Introduction

The selective catalytic reduction (SCR) of nitrogen oxides (NO_x_) with ammonia is one of the most efficient and widely applied technologies for industrial flue gas purification. In the SCR process, ammonia reacts selectively with NO_x_ in the presence of oxygen to produce harmless nitrogen and water. Enhancing the kinetic efficiency of SCR catalysts mainly depends on precise regulation of redox-active sites in catalytic materials. Metal doping (e.g., Fe- and Ce-based materials) and lattice defect engineering are widely used strategies to optimize catalytic performance [1,2], among which oxygen vacancy defects have received particular attention due to their capability to modulate the electronic structure and redox behavior [3,4].

Oxygen vacancies are coordinatively unsaturated sites that can modify the local charge distribution, promote oxygen mobility, and facilitate adsorption and activation of NO_x_ species. They play a key role in enhancing redox reactions during SCR processes. However, there remain two major challenges in current studies: first, the mechanism of how different preparation methods (e.g., heat treatment, hydrogen reduction, etc.) directionally generate various defect types (point, line, and plane) has not been fully clarified [3,4,5]; and second, it is difficult to achieve in situ quantitative characterization of oxygen vacancy concentration using conventional techniques such as EPR or XAFS [6,7,8].

Fly ash was selected as the catalyst substrate because it is abundant, inexpensive, and naturally rich in transition metals such as Fe and Al (Table 1). Its porous structure (FA initial specific surface area of 2.27 m^2^/g) offers an ideal support for defect engineering and significantly reduces the cost by 70% compared with commercial catalysts. In this work, an Oxygen Vacancy Index (*OVI*) is proposed as a quantitative descriptor of surface redox activity. *OVI* is calculated from the peak area ratio of defective oxides to lattice oxides in XPS O1s spectra (Area_Ononlattice_/Area_Olattice_), and its validity in fly ash-based catalytic systems has been verified through correlation with H_2_-TPR and O_2_-TPD data [9].

A combined process of wet slag magnetic separation, oxygen-enriched ball milling, and plasma discharge was used to regulate multi-scale defects. Magnetic separation enabled Fe enrichment (Fe_2_O_3_ content increased from 6.42 wt.% to 38.03 wt.%), ball milling generated surface defects (sevenfold increase in surface area), and plasma treatment introduced high-energy etching to form point and line defects (120% enhancement of *OVI*). This synergistic modulation strategy showed superior efficiency compared with single modification methods [3,4]. Furthermore, a quantitative model linking process parameters (magnetic separation intensity, ball milling time, discharge power) and *OVI* was established using the response surface method, providing a new approach for fly ash resource utilization. The optimized catalyst achieved a NO conversion of 85.58% at 400 °C, comparable to that of commercial V_2_O_5_-WO_3_/TiO_2_ catalysts [10].

**Figure 1:**
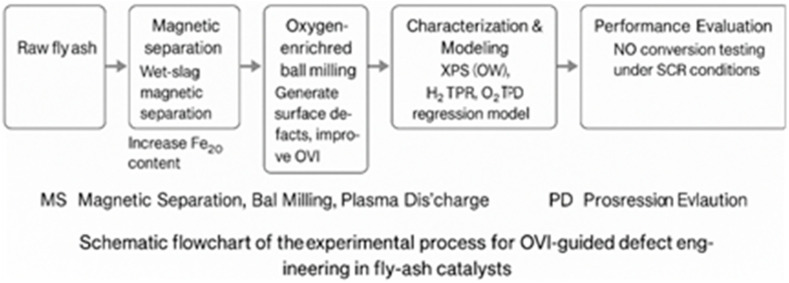
Flow chart.

The objectives of this study are to develop a quantitative *OVI*-based approach to describe oxygen vacancy defects in fly ash catalysts, establish the relationship between process parameters and *OVI*, and demonstrate that multi-scale defect engineering can enhance denitrification performance while maintaining low material cost. The flow chart is shown in Figure 1.

## 2. Materials and Methods

Fly ash was used as the catalyst for the experiments. The raw fly ash (FA) was sourced from Dalian Huaneng Power Plant (Dalian, China), and the fly ash sample was obtained from the electrostatic precipitator of the Dalian Huaneng Power Plant, which mainly uses bituminous coal as the fuel source, and its composition is accounted for as shown in Table 1.

The chemical composition of the fly ash was analyzed by XRF and FTIR to assess the presence of sulfur- and carbonate-related impurities. The results indicated that the sulfur oxide content was below 0.3 wt.%, while carbonate species were negligible. Such low levels are typical for bituminous-coal-derived fly ash and are not expected to significantly influence catalytic performance. Moreover, during the magnetic separation, ball milling, and plasma modification steps, possible surface sulfur species were effectively removed due to thermal desorption and plasma-induced decomposition. This conclusion is further supported by the absence of S 2p signals in the XPS spectra, confirming that the final materials are essentially free of sulfur contamination. Consequently, the influence of residual sulfur or carbonates on catalytic activity can be considered negligible under the tested SCR conditions.

### 2.1. Experimental Procedure

**Figure 2:**
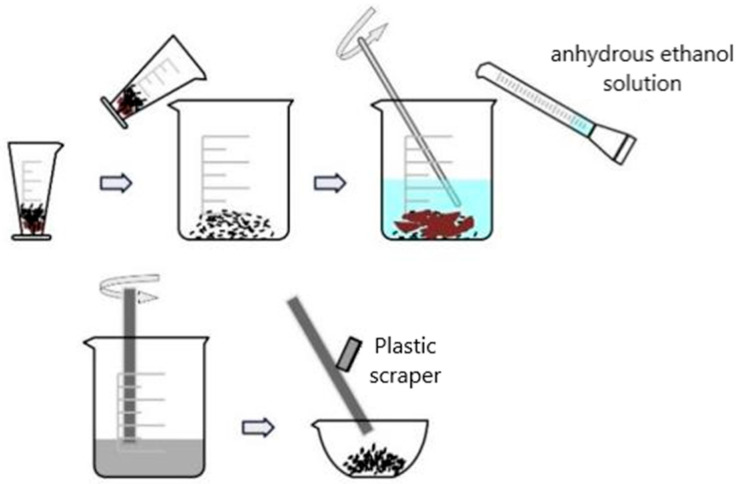
Wet slag magnetic separation flow diagram. (Black–brown particles: fly ash (MSFA); Light blue liquid: anhydrous ethanol solution/slurry; Grey liquid: FA slurry after magnetic stirring; Grey rod: magnetic bar/plastic scraper; Arrows: sequence of operation).

(1) Wet slag magnetic separation. After weighing a certain amount of FA, it was placed in a container, and an appropriate amount of deionized water was added in the ratio of water to material of 4:1. We added 3% anhydrous ethanol solution into the mixed slurry and mixed with an electronic mixer for about 4 min until the slurry was evenly distributed. We then put the magnetic bar into the mortar and slowly stirred in a circle for about 60 s. A plastic scraper was used to scrape off the magnetic mixture adsorbed on the surface of the magnetic bar, which was then put into a vessel for spare. Then it was put into the drying box at 45 °C; for 6 h until it was dried sufficiently to obtain the raw fly ash after magnetic separation (MSFA). The flow chart of this experimental process is shown in Figure 2.

(2) Oxygen-enriched ball milling. According to the grinding media diameter size 4 mm, 6 mm, 10 mm, 20 mm zirconia grinding balls according to the 2:3:3:2 mass ratio for the proportion, we chose the ball milling filling rate of 0.3, weighing 21.4 g of MSFA into the ball milling jar.

**Figure 3:**
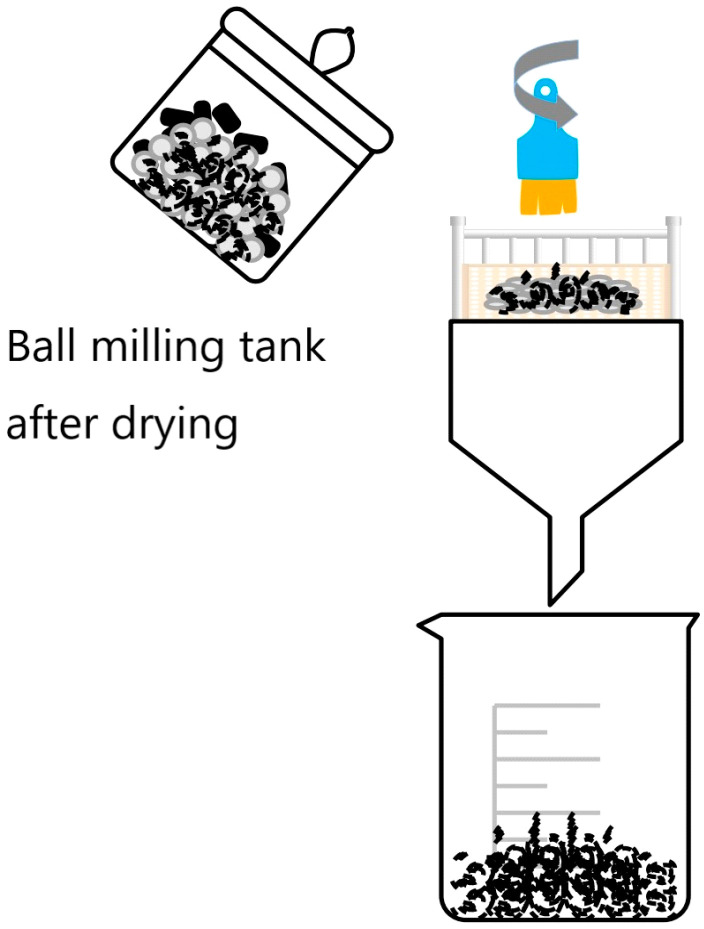
Oxygen-enriched ball milling process diagram. Black–grey particles represent the mixture of BMFA powder and zirconia grinding balls; the blue and orange component at the top represents the brush used to scrub the grinding balls; the light orange rectangular area represents the screen (sieve) used to separate BMFA from the grinding balls; the outlined containers represent the ball milling tank and the receiving beaker for the separated BMFA.

In each ball milling jar, 10 mL of 7.7% hydrogen peroxide solution for laboratory use was added, and the slurry in the ball milling jar appeared as a paste after addition, and the rotational speed of the ball mill was set at 550 rpm, and three groups of magnetically selected fly ash (BMFA) after ball milling were prepared according to different ball milling times. When the experiment was completed, the ball milling jars with the ash paste were dried in a drying oven at 45 °C for 8 h in a blast air until the ash paste was completely dry. After presenting the powder, the surface of the grinding balls was scrubbed with a brush, and the BMFA material and zirconia grinding balls were separated after being sieved through a screen, the flow diagram is shown in Figure 3, and the sealing material was kept in reserve.

**Figure 4:**
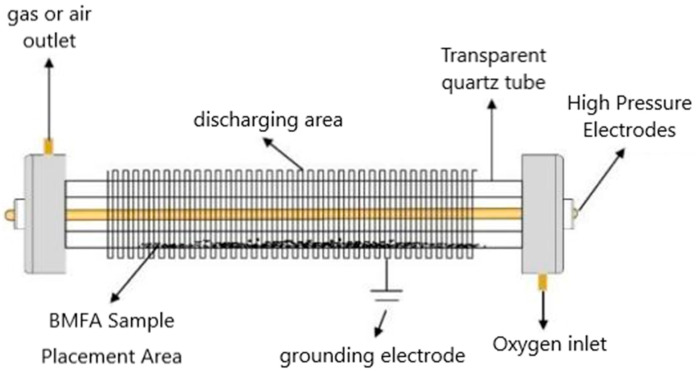
Schematic diagram of plasma discharge process.

(3) Plasma discharge. The discharge spacing was selected as 2 mm, the discharge length was 175 mm, the plasma reaction device was built as shown in Figure 4, and 5 g of BMFA material was evenly spread in the discharge area. Under the O_2_ atmosphere, the modification was carried out for 20 min, and the materials were taken to be prepared with different power to obtain the magnetically selected fly ash (PFA) modified by ball milling and plasma discharge.

### 2.2. Evaluation of Redox Properties

Oxygen vacancy defects significantly enhance the redox activity of catalytic materials by lowering the oxygen activation energy barrier (O_2_→O*) and optimizing the electronic state density of metal active centers (e.g., Fe^3^^+^/Fe^2^^+^ redox pairs) [5,10,11,12]. Oxygen vacancies in Mo-doped Co_3_O_4_ significantly enhance the electrocatalytic performance by modulating Co^3^^+^/Co^2^^+^ oxidation state and promoting intermediate adsorption to significantly enhance the electrocatalytic performance [5]. Oxygen vacancies in the crystalline surface of CeO_2_{111} significantly enhance the SCR reaction efficiency by promoting the oxidation of NO to NO_2_ [12]. In this study, we have further verified through *OVI* quantitative modeling that the oxygen vacancies have a significant effect on the denitrification performance. regulation of denitrification performance. Currently there is not a recognized method for how to quantify and identify oxygen vacancies. The usual methods are electron paramagnetic resonance (EPR) spectroscopy [6], synchrotron radiation-based X-ray absorption fine structure (XAFS) spectroscopy [7], positron annihilation lifetime spectroscopy (PALS) [8], Raman spectroscopic analysis [13], scanning transmission electron microscopy (STEM) [14] and X-ray photoelectron spectroscopy (XPS) [15]. The redox capability of a catalyst is a crucial indicator of its ability to activate oxygen species and participate in oxidation-reduction cycles. In the context of fly ash-based materials, such redox behavior is mainly governed by the generation and migration of oxygen vacancies, which alter the electronic structure and surface activity of metal sites. Therefore, evaluating the oxygen vacancy concentration provides a direct insight into the intrinsic redox capacity of the material.

In this study, the Oxygen Vacancy Index (*OVI*) was proposed as a quantitative descriptor to evaluate the redox property of the catalyst. The *OVI* was determined from the XPS-O1s spectra according to the peak area ratio of defective oxides (531–532.3 eV) to lattice oxides (529.7–530.1 eV). Compared with conventional methods such as EPR and XAFS, XPS offers better surface sensitivity (<10 nm) and enables simultaneous identification of oxidation states (e.g., Fe^2^^+^/Fe^3^^+^). The correlation between *OVI* and H_2_-TPR results (R^2^ = 0.91) further confirms that *OVI* can effectively reflect the material’s redox performance, thus providing a reliable quantitative indicator for assessing oxygen vacancy density and redox activity.

For fly ash-based non-homogeneous material properties, XPS is more suitable for quantitative oxygen vacancy characterization than EPR (detection of paramagnetic defects only) and XAFS (requiring highly homogeneous samples) due to its surface sensitivity (detection depth < 10 nm) and chemical state resolution (e.g., Fe^2^^+^/Fe^3^^+^ valence state analysis) [6,15]. In addition, the peak area ratio (Area_Ononlattice_/Area_Olattice_) of defective oxides (531–532.3 eV) to lattice oxides (529.7–530.1 eV) in the XPS-O1s spectra has been shown to have a significant positive correlation with the H_2_-TPR reduced peak area (*R*^2^ = 0.91, the literature [9]), which provides a theoretical basis for the present study to *OVI* indicators were constructed to provide theoretical basis.

In the XPS spectra, the oxygen O1s spectral fit generally has two peaks, O1sA and O1sB, representing two different surface substances. O1sA with binding energies in the range of 529.7–530.1 eV belongs to lattice oxides, while O1sB with binding energies in the range of 531–532.3 eV belongs to defective oxides, and the O1sB/(O1sA + O1sB) peak area ratio indirectly responds to the relative concentration of oxygen vacancies on the catalyst surface [9]. Specifically, the higher ratio of the intensity of the peak of oxygen vacancies (Area_Ononlattice_) to the intensity of the chemical bonding peak (Area_Olattice_) indicates the higher number of oxygen vacancies. The greater the ratio of the peak area of the oxygen vacancy peaks to the peak area of the chemical bonding peaks, the stronger the intensity of the oxygen vacancy peaks.

Based on the XPS-O1s split-peak fitting results (Gaussian–Lorentzian function, half-height-width constraint ± 0.2 eV), the Oxygen Vacancy Index (*OVI* = Area_Ononlattice_/(Area_Ononlattice_ + Area_Olattice_) × 100%) was defined, and its calibration process was performed by comparing the H_2_ consumption of the H_2_-TPR [2] (e.g., *OVI* = 80.59% corresponds to H_2_ consumption of 2.7 mmol/g). This metric quantitatively characterizes the oxygen vacancy density per unit surface area and is more reproducible than traditional qualitative methods (e.g., STEM defect counting) [9,15]. 

It should be noted that the XPS-*OVI* method is sensitive to surface carbon contamination (the C1s peak needs to be corrected to 284.8 eV) and cannot distinguish between oxygen vacancies and hydroxyl oxygen contributions. Follow-up studies can further resolve the local electronic structure of oxygen vacancies by synchrotron radiation soft X-ray absorption spectroscopy (sXAS) [7].

### 2.3. Characterization Methods

X-ray fluorescence spectrometry (XRF) analysis was performed using a Axios sequential X-ray fluorescence spectrometer (Malvern Panalytical B.V., Almelo, The Netherlands. Scanning electron microscope (SEM) observation using the Merlin Compact field-emission scanning electron microscope (Carl Zeiss AG, Oberkochen, Germany), the powdered material directly on the conductive adhesive sample making, observation using the scale were 1 μm, 2 μm, 10 μm. N_2_-physisorption analysis was performed using a fully automated surface and porosity analyzer (Quantachrome Nova 2000e, Quantachrome, Boynton Beach, FL, USA). The Brunauer–Emmet–Teller (BET) model was applied to calculate the specific surface area, while the Barrett–Joyner–Halenda (BJH) method was used to determine the total pore volume and average pore size. The degassing temperature was set at 120 °C for 6 h before testing. Ultima IV multipurpose X-ray diffractometer (Rigaku Corporation, Tokyo, Japan) analysis was conducted using a Rigaku Ultima IV diffractometer equipped with a Cu Kα X-ray source (λ = 0.15406 nm). The scan rate was set at 2°/min with a 2θ range of 10°–80°. ESCALAB 250Xi X-ray photoelectron spectrometer (Thermo Fisher Scientific, Waltham, MA, USA) was carried out using an American Thermo Electron Spectroscopy (TES). Analysis was selected from the American Thermo Scientific ESCALAB 250Xi spectrometer, which was used to carry out the original data of peak fitting calculation. Nicolet iS20 FTIR spectrometer (Thermo Fisher Scientific, Waltham, MA, USA) analysis was performed using a Thermo Scientific Nicolet iS20 analyzer, USA. JEM-2100F transmission electron microscope (JEOL Ltd., Akishima, Tokyo, Japan) images were used to analyze the surface of the materials at a scale of 1 μm to 5 nm using the film sampling method, respectively.

## 3. Results and Analysis

### 3.1. Response Surface Method Design of Experiments

Using Box–Behnken method to design the experiment, referring to the relevant information [16] and the best experimental results of the same group [17,18], three significant influencing factors were selected: magnetic separation intensity, ball milling time and discharge power, and three levels were set for each of these factors, as shown in Table 2. 

According to Table 2, the Oxygen Vacancy Index (*OVI*) was used as the evaluation parameter. For each test, 0.3 g of the final PFA sample was analyzed by XPS. The O1s spectra were fitted using Thermo Avantage software (version 5.x; Thermo Fisher Scientific, Waltham, MA, USA), where the ratio of the oxygen vacancy peak area to the lattice oxygen peak area was used to calculate the *OVI*. In total, 17 experiments were conducted, and the corresponding results are summarized in Table 3.

According to Table 3, the *OVI* values of the center point experiments (Run 1, 8, 13, 15, and 16) were 80.59%, 79.59%, 78.94%, 83.61%, and 80.79%, respectively, with a standard deviation of ±1.2% and a relative standard deviation (*RSD*) of 1.49%, which indicated that the experiments were well reproducible (*RSD* < 5%). This verifies the reliability of the response surface method in the optimization of process parameters and provides a data basis for the subsequent model construction.

The quadratic regression equation ANOVA of the experimental results is shown in Table 4. After analyzing the results of the above 17 groups of PFA data materials using Design-expert software (version 13; Stat-Ease, Inc., Minneapolis, MN, USA), the model *F*-value is 3.90, and the established regression overall model has a *p*-value of <0.05 and a *p*-value of the misfit term of >0.01, which proves that the above response surface model is established. The obtained regression equation is as follows:

*OVI* = 80.70 − 0.1162 × A + 2.28 × B + 1.08 × C − 1.28 × AB + 0.3775 × AC + 2.46 × BC − 4.32 × A2 + 0.4192 × B2 − 5.35 × C2

and the goodness of fit R^2^ = 0.8336, which is relatively close to 1. Therefore, it can be judged that the regression model is fit better.

Among them, the primary term coefficient can characterize the main effect, indicating the linear effect of factors A, B, and C on the response; the secondary term coefficient can characterize the interaction, indicating the nonlinear mutual effect of factors AB, AC, and BC on the response; and the square term coefficient can characterize the nonlinear effect of factors A^2^, B^2^, and C^2^ on the response.

*F*-value and *p*-value in ANOVA are important indicators used to determine whether there is a significant difference between the means of different groups, *F*-value reflects the size of the difference between the groups, while the *p*-value helps to determine whether such a difference is significant or not, and the smaller the *p*-value indicates the more significant. Therefore, the order of the size of the influence of the three factors is as follows: oxygen-enriched ball milling time > plasma discharge power > wet slag magnetic separation magnetic field strength.

**Figure 5:**
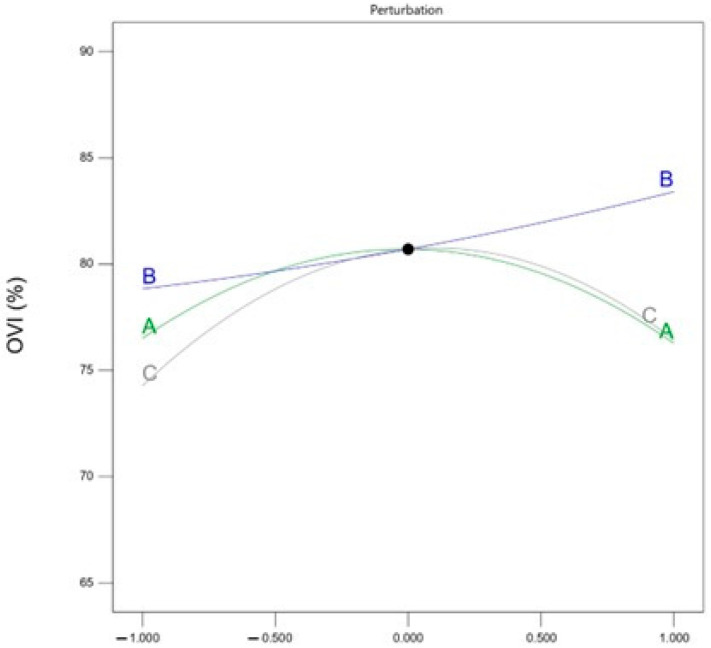
Comparison plot of factors affecting the oxygen vacancy factor of PFA (A: magnetic separation intensity; B: ball milling time; C: discharge power; R1: *OVI*).

Figure 5 shows the trend of *OVI* of PFA material affected by three factors. With the increase in magnetic separation intensity and discharge power, the *OVI* shows a trend of increasing and then decreasing; while the *OVI* is positively and proportionally correlated with the oxygen-enriched ball milling time.

### 3.2. Denitrification Experimental Design and Results

**Figure 6:**
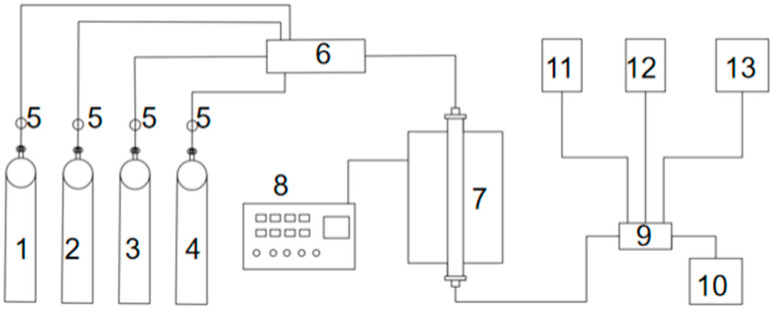
Schematic diagram of the selective catalytic reduction (SCR) denitrification setup. 1—NO gas cylinder; 2—N_2_ gas cylinder; 3—NH_3_ gas cylinder; 4—O_2_ gas cylinder; 5—mass flow meter; 6—gas mixing tube; 7—fixed-bed reactor with tube furnace; 8—temperature controller; 9—gas detector tube; 10—tail-gas absorber; 11—NH_3_ sensor; 12—NO sensor; 13—temperature and humidity detector.

The experimental setup used for the denitrification experiment is shown in Figure 6.

The concentrations of NO and NH_3_ in the outlet gas were continuously monitored using electrochemical sensors (Model: Testo 350, Testo SE & Co. KGaA, Grünwald, Germany). The detection limits for NO and NH_3_ were 1 ppm, and the measurement accuracy was ±2% of the reading. The sensors were calibrated before each test to ensure data reliability.

The reaction temperatures selected for the experiment were 100 °C, 200 °C, 300 °C and 400 °C, and the reaction time at the set temperature was 30 min. In the experiment, 4 g each of FA and PFA were used. Denitrification results are shown in Table 5. Table 5 shows the temperature-dependent NO conversion; the enhanced activity confirms that multi-scale defects promote low-temperature SCR. The denitrification experiments in the table were repeated three times with an error of ±1.5% in NO conversion. PFA showed higher NO conversion than FA.

By comparing the catalytic effect of FA and PFA, it can be found that the modified nano fly ash shows higher performance in denitrification efficiency. This indicates that the denitrification efficiency increased with the increase in redox properties.

### 3.3. Material Characterization

#### 3.3.1. XRF Analysis 

The compositional content and elemental content variations in FA, MSFA, BMFA and PFA were determined using X-ray fluorescence spectrometer analyzer and the results are shown in Table 6 (Table 6 lists only the major oxide components (SiO_2_, Al_2_O_3_, Fe_2_O_3_, CaO, K_2_O, TiO_2_) determined by XRF for each sample. Trace oxides (e.g., MgO, Na_2_O, SO_3_) and other minor constituents are not included) and Table 7.

As can be seen from the changes in the composition of the samples, magnetic separation substantially reduces the SiO_2_ content and greatly increases the Fe_2_O_3_ content, while the other major components remain largely unchanged. After the subsequent oxygen-enriched ball milling and plasma discharge modifications, the overall chemical composition does not change significantly.

From the changes in elemental content presented in Table 7, it is evident that the majority of the oxygen content originates from SiO_2_ and Fe_2_O_3_. Because the SiO_2_ content decreases after magnetic separation, the total O content remains nearly constant at that stage. Furthermore, in the magnetically separated sample (MSFA), the Fe content surpasses the Si content, becoming the most abundant element. This pronounced Fe enrichment provides a substrate with excellent potential for the preparation of high-redox-performance materials [19].

#### 3.3.2. SEM Analysis

SEM characterization was carried out for the materials at each experimental stage to observe the surface morphology characteristics.

**Figure 7:**
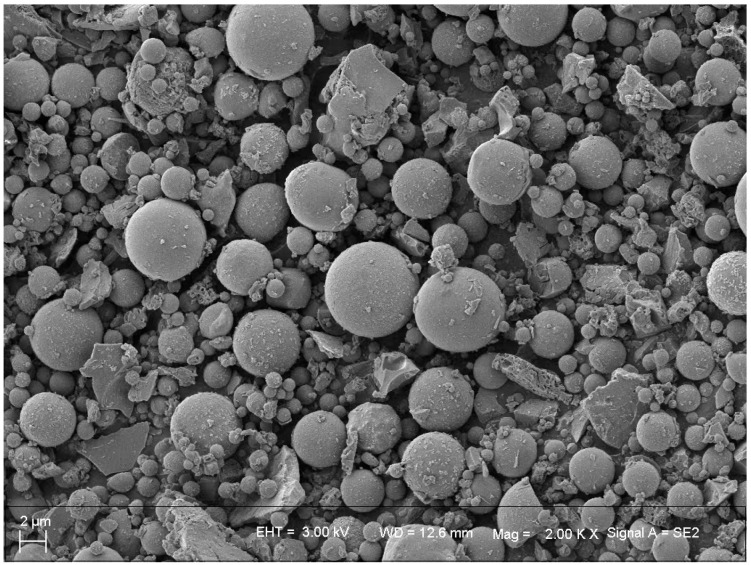
SEM graph of FA.

The SEM image of the FA substrate used for the experiments is shown in Figure 7.

Most of the particles in FA are mainly spherical, and it is known from the XRF and XRD analyses that these spherical particles are mainly composed of vitreous particles in the molten state after high-temperature sintering, with varying diameters and sizes, but most of them are more regular in shape.

**Figure 8:**
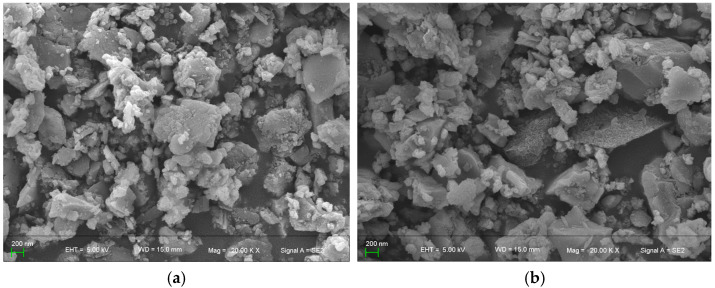
SEM graph of BMFA (**a**) and PFA (**b**).

The SEM images of BMFA and PFA are shown in Figure 8. In Figure 8, BMFA shows a particle size that is at least 10 times smaller than the original material. The reduced particle size may lead to increased surface area and higher reactivity. The material particles also underwent a shape change after the oxygen-enriched ball milling process, gradually changing from the original near-spherical shape to spherical particles, and the particles no longer showed a spherical shape but an approximate flocculent structure.

The SEM images of the surface of PFA presented an increase in surface roughness and an increase in the surface microporosity content, which microscopically indicated the formation of nanoparticle aggregates, nanowires or nanostructures, and micro- and nano-features on the surface. In addition, it can be observed that the melted or dissolved regions present features such as being smooth, flat or uneven, which are arranged in a disordered manner to form a small number of inter-particle channels.

#### 3.3.3. BET Analysis

The Brunauer–Emmet–Teller (BET) method was used to evaluate the specific surface area and pore structure of the samples. These parameters provide important insights into the influence of different modification processes on the surface reactivity and gas adsorption behavior of the catalysts. Table 8 was obtained by BET analysis.

As can be seen from Table 8, after a series of processing, the specific surface area of the material was enhanced from 2.3 m^2^/g in FA to 16.0 m^2^/g in PFA, which is nearly seven times, and the total pore volume of the material at each stage was also enhanced by an order of magnitude, and the average pore diameter was also enlarged from 9.16 nm in FA to 19.6 nm in PFA due to the processing process at each stage. Both the enlarged pore size and pore volume proved that this set of processing procedures provided FA with a loose and porous micro-morphology that could be used to enhance the oxygen vacancy content of the redox properties.

**Figure 9:**
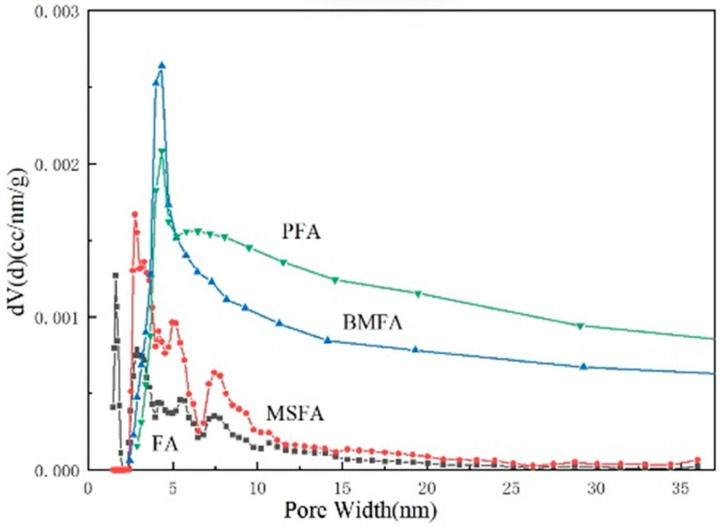
Distribution of microporous pore size of fly ash at each stage (DFT).

Figure 9 shows the pore size distribution of fly ash micropores at each stage. The trend of the microporous pore size distribution of the materials at each stage can also be visualized from the figure, and the average particle size curve has a tendency to flatten to the right side after each high redox treatment, in which the final material, PFA, possesses the highest specific surface area, total pore volume, and average pore size at the same time, which suggests that the PFA material contains a large number of microporous structures.

#### 3.3.4. XRD Analysis

**Figure 10:**
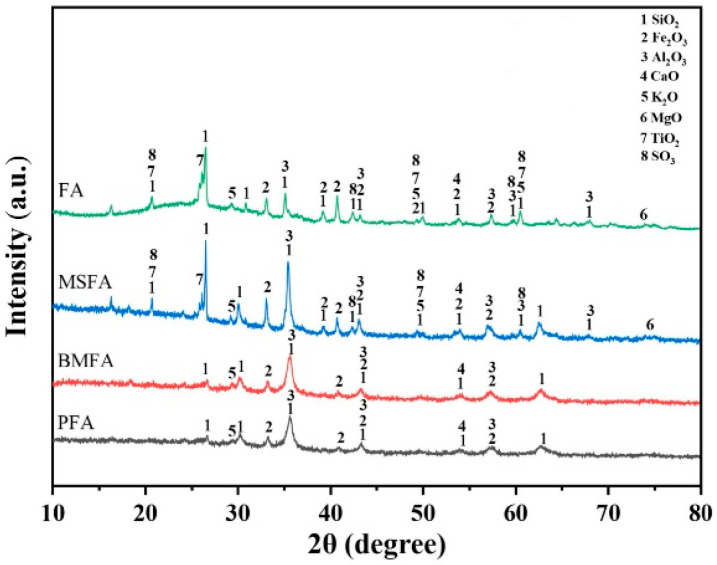
XRD patterns of FA, MSFA, BMFA and PFA. Phase identification was performed using standard reference data from the ICDD PDF-2 database (2022).

The XRD patterns of FA, MSFA, BMFA, and PFA are shown in Figure 10. In both the raw fly ash (FA) and the magnetically separated fly ash (MSFA), the major crystalline phases are identified as quartz (SiO_2_, PDF#74-1811) and mullite (Al_6_Si_2_O_13_, PDF#82-1237), consistent with the XRF results indicating SiO_2_ and Al_2_O_3_ as the primary components. After magnetic separation, the MSFA pattern shows significantly enhanced peaks associated with iron oxide phases. For example, new or intensified peaks appear at 2θ ≈ 35.7°, 40.9°, and 57.6°, which can be indexed to hematite (α-Fe_2_O_3_, PDF#33-0664). These correspond to hematite reflections (e.g., the (104)/(110), (113), and (018) planes) and confirm an increase in the hematite content in MSFA compared to FA. Quartz and mullite remain the dominant phases in MSFA (with strong reflections at ~26.6° from quartz and ~16.4°, 25.9° from mullite), but hematite becomes a prominent secondary phase.

Notably, a weak diffraction peak was observed around 2θ ≈ 18.0° in both the FA and MSFA patterns. This peak was previously misattributed to “amorphous components,” but amorphous phases produce a broad hump in XRD rather than a sharp reflection. We have re-assigned the ~18° feature to a minor crystalline constituent of the fly ash: magnetite (Fe_3_O_4_, PDF#19-0629). The presence of magnetite (or its structurally similar form maghemite, γ-Fe_2_O_3_) is indeed common in coal fly ash. Assigning the ~18° peak to magnetite clarifies that this reflection originates from a crystalline iron-oxide phase rather than an amorphous phase. In summary, magnetic separation enriches the sample in iron oxides – primarily hematite (with minor magnetite) – while the principal aluminosilicate phases (quartz and mullite) remain unchanged.

After the oxygen-enriched ball-milling treatment, the XRD pattern of BMFA (ball-milled fly ash) shows that the intensities of all diffraction peaks decrease and their widths increase markedly, compared to MSFA. In particular, the hematite peak at ~35.7° (now the most intense peak, PDF#33-0664) becomes the main crystalline component in BMFA. The broadening and weakening of peaks (for all phases, including hematite, mullite, and quartz) after ball milling indicate a reduction in crystallite size and partial loss of long-range order due to mechanical milling. Using Scherrer’s formula, the crystallite size of hematite (from the 35.7° peak) is estimated to decrease from about 52.3 nm in FA to 18.7 nm in BMFA, confirming that the high-energy milling significantly refined the grain size. The reduction in crystallite size (and corresponding increase in peak width) is consistent with the 7-fold BET surface area increase, and it provides a structural basis for the generation of surface defects (e.g., grain boundary dislocations) during ball milling. The diffraction peaks of mullite and quartz in BMFA are greatly diminished and broadened; the appearance of an elevated baseline (“burrs”) in the 20–40° region suggests that many Si–O and Al–O bonds were broken, and some crystalline material was rendered X-ray amorphous. Overall, oxygen-enriched ball milling substantially reduces the particle size, disrupts the crystal lattices of the aluminosilicates, and increases the specific surface area of the material.

Compared with BMFA, the plasma-treated sample (PFA) shows a slight shift of the hematite peak from 35.726° to ~35.625°, which we attribute to lattice distortion in hematite caused by the creation of oxygen vacancies during the plasma discharge process. The generation of oxygen vacancies (Fe_2_O_3_) and the expulsion of some lattice oxygen can subtly alter the hematite lattice parameters, hence the small peak shift. Additionally, the plasma’s high-energy ion bombardment likely disperses some residual impurities into finer particles. As a result, the high-2θ end of the PFA XRD pattern becomes smoother (lower background and noise), indicating a further reduction in crystalline domain size and possibly an increase in amorphous content. In summary, each sequential treatment (magnetic separation, ball milling, plasma discharge) modifies the phase composition and crystallinity of the fly ash: magnetic separation enriches crystalline hematite (and magnetite) content, ball milling diminishes crystallite size and crystallinity, and plasma treatment induces lattice defects (oxygen vacancies) in the iron oxide phase, all of which contribute to the enhanced redox properties of the final PFA material.

#### 3.3.5. XPS Analysis

The collected FA, MSFA, BMFA and PFA powder materials were tested by conventional XPS to obtain the photoelectron spectra in the O1s energy region, and then Avantage software (version 5.x; Thermo Fisher Scientific, Waltham, MA, USA) was used to obtain the O1s peak fitting spectra.

**Figure 11:**
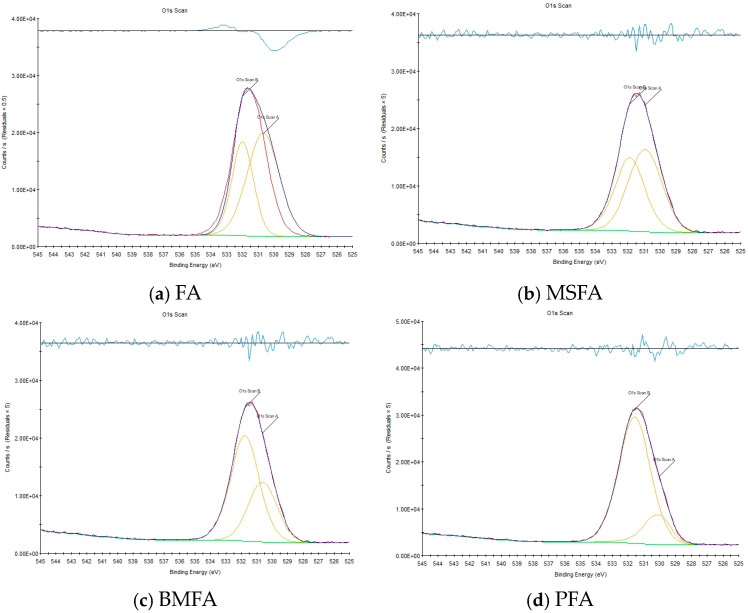
XPS plots of O 1s split peak fits: (**a**) FA; (**b**) MSFA; (**c**) BMFA; and (**d**) PFA.

The XPS test results are shown in Figure 11.

Figure 11a–d show the O1s peak fitting results of FA, MSFA, BMFA, and PFA, respectively, and their quantified *OVI*s are 38.04%, 44.97%, 64.29%, and 83.61%, respectively. The wet slag magnetic separation, oxygen-enriched ball milling, and plasma discharge machining processes represented by each material enhanced the *OVI* of the material by 18.22%, 42.96%, and 30.05%, respectively, with the final result being a 120% enhancement over the initial value.

Based on the XPS analysis, it can be obtained that among the three processing methods, the oxygen-enriched ball milling process contributes the most to the enhancement of *OVI*, indicating that the ball milling leads to a significant increase in the specific surface area of the MSFA material, while hydrogen peroxide acts as an additive to provide hydroxyl groups, which further promotes the generation of surface defects. The plasma discharge modification process has the second largest contribution to the enhancement of *OVI*, probably due to the breakage and reorganization of surface chemical bonds caused by the collisional excitation of high-energy electrons and ions. These fracture and reorganization processes cause the dissociation and rearrangement of oxygen atoms, which increases the number of oxygen vacancies, resulting in the enhancement of *OVI*.

#### 3.3.6. FTIR Analysis

**Figure 12:**
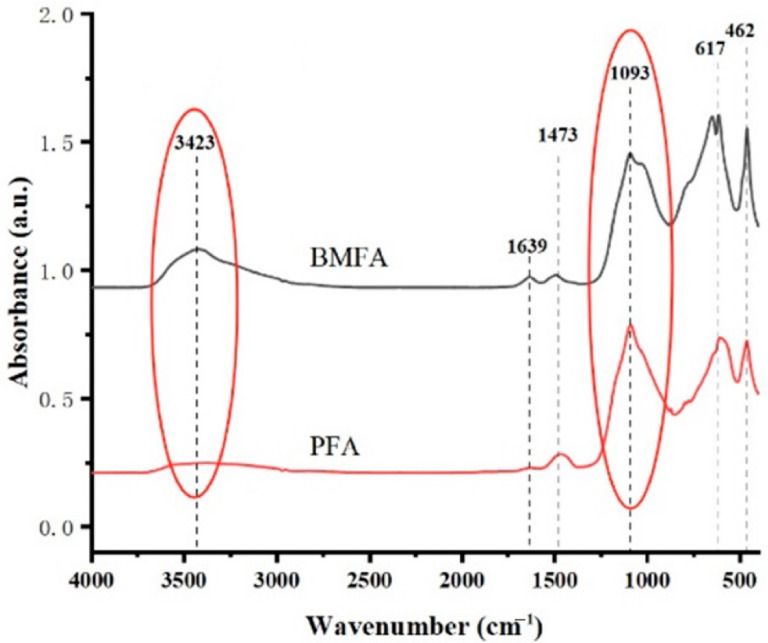
FTIR profiles of BMFA and PFA.

Figure 12 shows the FTIR patterns of BMFA and PFA. From the FTIR diagram of BMFA, it can be seen that the telescopic vibration absorption peak of the hydroxyl (O-H) bond at 3423 cm^−1^; the telescopic vibration peak of the carbonyl (C=O) bond at 1630 cm^−1^; the bending vibration peak of the C-H bond at 1493 cm^−1^ corresponds to the C-H bond of the aromatic compounds; and the telescopic vibration peak of the C-O bond at 1093 cm^−1^, which may indicate the functional groups of alcohols, ethers, or functional groups such as carboxylic acids; at 654 cm^−1^ and 617 cm^−1^ correspond to inorganic substances in the fly ash, such as oxides; and at 462 cm^−1^ usually corresponds to the vibrations of metal oxides, such as the vibrations of Fe-O or Al-O bonds.

From the FTIR diagram of PFA, it can be seen that the intensity of the absorption peak of the telescopic vibration corresponding to the hydroxyl (O-H) bond at 3485 cm^−1^ is significantly weakened, and since the hydroxyl functional group is usually bonded to oxygen atoms, the reduction in hydroxyl reflects the reduction in the oxygen content in the material. The peak corresponding to the location of the carboxyl group (C=O) disappeared. The bending vibration peak at 1473 cm^−1^ corresponding to the C-H bond indicates the presence of methyl (-CH_3_) or methyl groups, which is speculated to be a possible result of the organic residue in the material or the surface modification. The telescopic vibration peak at 1093 cm^−1^ is the one for the C-O bond, which indicates the functional groups of alcohols, ethers, or carboxylic acids; the peaks at 606 cm^−1^ and 465 cm^−1^ are significantly weakened peaks, which usually correspond to the vibration of inorganic substances in the material, and the weakening of the intensity of these peaks indicates that the oxygen content in the material is reduced.

#### 3.3.7. TEM Analysis

**Figure 13:**
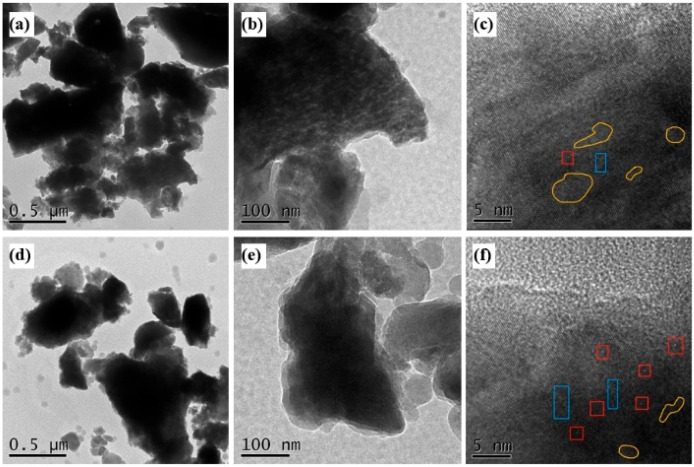
HR-TEM graph of PFA and BMFA. (**a**) TEM micrograph of PFA at low magnification (scale bar: 0.5 μm) showing irregularly shaped particles; (**b**) Higher-magnification TEM image of PFA (scale bar: 100 nm); (**c**) HR-TEM image of PFA (scale bar: 5 nm) highlighting dot defects marked by red boxes and line defects indicated by blue boxes; (**d**) TEM micrograph of BMFA at low magnification (scale bar: 0.5 μm); (**e**) Higher-magnification TEM image of BMFA (scale bar: 100 nm); (**f**) HR-TEM image of BMFA (scale bar: 10 nm) showing dot defects (red boxes), line defects (blue boxes) and irregular surface defects circled in yellow. The red boxes indicate rounded dot-like defects caused by oxygen vacancies or plasma discharge treatment. The blue boxes highlight line defects (continuous dark lines) produced by ball-milling and plasma discharge. The yellow outlines mark surface defects/facets where lattice fringes are not visible due to enhanced electron scattering and absorption.

The HR-TEM images of BMFA and PFA are shown in Figure 13. The surface of BMFA shows rounded dot bright vacancies, and the content of dot defects has been enhanced after plasma discharge modification (marked by red boxes), which is mentioned in some studies [20,21,22]. Line defects generally show up as continuous dark lines in TEM images, which is because oxygen vacancies cause distortions in the local atomic structure, leading to the blockage of electron transmission, which is thus presented as dark regions in the image [23]. Therefore, the blue boxed areas in Figure 13c,f can be recognized as line defects produced by ball milling and plasma discharge modification. Facet defects often appear as dark regions in TEM images and lattice fringes cannot be visualized in them, which is due to the local distortion of the lattice at the defects leading to enhanced scattering and absorption of electrons, which produces attenuated signals in the microscope images [24]. Such dark regions can sometimes present irregular shapes depending on the defect type and morphology. The regions circled by the yellow lines can be clearly seen in Figure 13c,f as conforming to the morphology of the surface defects.

## 4. Discussion

The ANOVA of the results of orthogonal experiments showed that the oxygen-enriched ball milling time and plasma discharge power had a significant effect on *OVI*. The effects of these two factors on the adsorption pore size and the content of oxygen groups were investigated, and the ANOVA results are shown in Table 8 and Table 9. Where oxy groups include functional groups of alcohols or phenols with inorganic oxide groups. B and C refer to the oxygen-enriched ball milling time and plasma discharge modification power, respectively.

In Table 9, the model is significant at 0.01 level. From the *p*-value observations, the oxygen-enriched ball milling time has a significant effect on the adsorption pore size. Therefore, increasing the oxygen-enriched ball milling time can simultaneously increase the adsorption pore size.

In Table 10, the model is significant at 0.05 level. From the *p*-value observation, the plasma discharge power has a significant effect on the oxygen group content. Therefore, proper increase in discharge power can reduce the oxygen group content.

**Figure 14:**
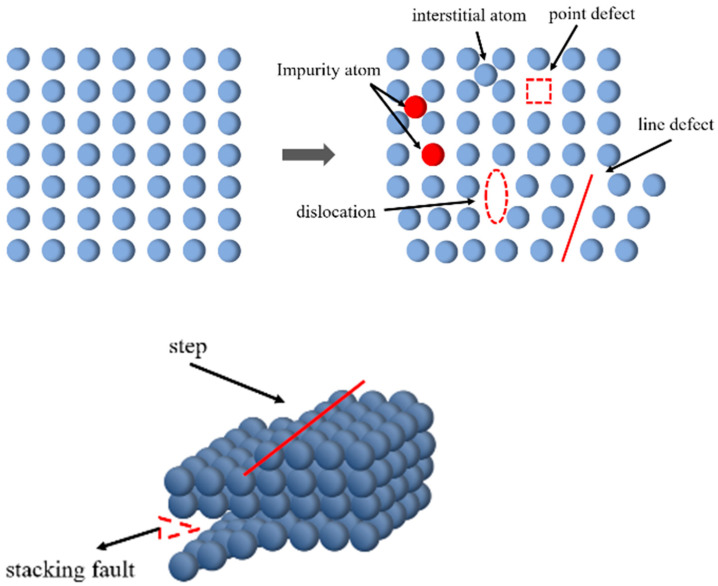
Schematic formation of point defects, line defects (**top**) and surface defects (**bottom**) in oxygen vacancies.

In Figure 13, BMFA has more face defects, while PFA has more point defects and line defects. It indicates that the oxygen-enriched ball milling method makes the material produce more face defects by inducing changes in the surface morphology and lattice structure of the material, especially increasing the specific surface area and decreasing the adsorption pore size. While plasma discharge modification produces an etching effect, when the plasma discharge power increases, the content of oxygen groups decreases, and the material will be more prone to fractures or dislocations, or even the absence of atoms or ions, thus producing more line defects and point defects. The formation of point defects, line defects and surface defects are shown schematically in Figure 14.

Facet defects significantly promote the physical adsorption of NH_3_ molecules on the catalyst surface by increasing the specific surface area (7-fold enhancement in BET) and pore structure (pore size enlarged to 19.6 nm) (6% enhancement of NO conversion at 100 °C for PFA in Table 5). And the dot/line defects enhanced the SCR reaction activity at low temperatures (NO conversion enhancement at 200 °C) by optimizing the electron transfer efficiency of Fe^3^^+^/Fe^2^^+^ redox pairs (18% enhancement of Fe^2^^+^ share as shown by XPS) and lowering the oxygen activation energy barrier (O_2_→O*) [Table 5], 5.83%) [19]. This synergistic effect allows PFA to maintain efficient denitrification performance over a wide temperature range (100–400 °C).

The linear regression relationship between the intensity of the oxygen group peaks of the summarized FTIR, the pore size data of the materials obtained from the BET test and the *OVI* is shown in Table 11.

The results showed that the pore size of PFA increased substantially compared to the average pore size of 9.1 nm presented by FA, and the oxygen group content of PFA also decreased significantly after co-modification compared to the oxygen group content of 78.9 a.u presented by FA. Regression analysis was performed based on the data in Table 10, and the results of linear regression analysis were obtained as shown in Table 12.

The *R*^2^ of the model was 0.692, which indicated that the oxygen groups and pore size together explained 69.2% of the change in *OVI*. And the model passed the F-test with an F-statistic value of 15.708, corresponding to a *p*-value of <0.05, indicating that the model as a whole is statistically significant. The VIF values of all independent variables are less than 5, indicating that there is no serious covariance problem. The DW value is around the number 2, indicating that the model does not have autocorrelation, and there is no correlation between the sample data, and the model is better.

Combined with the ANOVA in Table 9 and Table 10 and the characterization results in Section 3.3.3 and Section 3.3.6, the adsorbed pore size explains the role of face defects, while the oxygen group explains the role of point defects and line defects.

To obtain the proportions of the different effects, the absolute values of the normalization coefficients in Table 12 were calculated and then divided by the sum of their absolute values (0.952). The proportion of adsorbed pore size is 0.448, indicating that adsorbed pore size has 44.8% of the effect on *OVI* production. The percentage of residual oxygen groups is 0.504, indicating that oxygen groups have 50.4% of the effect on *OVI*. Since the linear regression analysis model only explains 69.2% of the variance, these proportions need to be multiplied by 0.692 to obtain values of 31 and 34.88, indicating that 31% of the 69.2% explanation is due to surface defects, while 34.88% is due to point and line defects. In addition, there is more than 69.2% unexplained variation in the *OVI*, i.e., 100% − 69.2% = 30.8%, which can be partially attributed to elemental variations due to wet slag magnetic separation, suggesting that elements possessing a variety of variable valence states can provide additional oxygen vacancies. Considering that the oxygen vacancies obtained by characterization analysis are all obtained by physical methods, the formation of defective oxides is dominated by physical mechanisms. The extra part, on the other hand, with high probability belongs to the lattice oxides, which are produced by chemical mechanisms due to the changes in the material elements themselves.

Compared with the commercial V_2_O_5_-WO_3_/TiO_2_ catalyst (88.2% NO conversion at 400 °C [8]), the performance difference between the fly ash-based materials of the present study (85.58% PFA conversion) is only 2.62% at similar temperature, but the feedstock cost is reduced by 70% (the cost of the commercial catalyst is about 3200 Yuan/ton, and the cost of the PFA is 960 Yuan/ton). In addition, the activity temperature window of PFA (100–400 °C) is 100 °C wider than that of commercial catalysts (200–450 °C), which is of great significance for denitrification of industrial flue gases at low temperatures. In the future, it is expected to further enhance its performance to commercial level by doping rare earth elements (e.g., Ce, La) or optimizing the carrier structure.

## 5. Conclusions

This work establishes an Oxygen Vacancy Index (*OVI*) as a quantitative indicator to guide defect engineering in fly ash-based catalysts. Using a multi-process approach combining magnetic separation, oxygen-enriched ball milling, and plasma discharge, the study achieved controllable oxygen vacancy formation and improved catalytic performance. The OVI model links process parameters, defect types, and denitrification efficiency, offering a practical method for evaluating and optimizing redox activity.

The proposed strategy highlights the practical value of cost reduction (≈70%), waste reuse, and industrial feasibility for large-scale NO_x_ control. The *OVI*-guided design provides a simple, low-cost pathway for transforming industrial solid wastes into high-performance catalysts.

Workflow summary: material design (fly ash activation) → structure regulation (multi-scale defects) → performance evaluation (NO conversion) → implication (industrial application and sustainability).

## Figures and Tables

**Table 1:** Main composition of fly ash content tables.

No.	Constituent	Content/%
1	SiO_2_	51.53
2	Al_2_O_3_	27.89
3	Fe_2_O_3_	6.42
4	CaO	5.95
5	TiO_2_	1.11
6	K_2_O	1.29

**Table 2:** Factors and levels.

Level	Factor A (GS)	Factor B (h)	Factor C (W)
1	8000	5	70
2	10,000	6.5	100
3	12,000	8	130

**Table 3:** Box–Behnken response surface experimental design and results for the preparation process of high redox nanoscale fly ash.

Run	Factor A (GS)	Factor B (h)	Factor C (W)	Response *OVI* (%)
1	10,000	6.5	100	80.59
2	12,000	6.5	130	69.36
3	8000	6.5	130	72.82
4	12,000	6.5	70	68.49
5	10,000	8	130	81.79
6	8000	8	100	79.32
7	8000	6.5	70	73.46
8	10,000	6.5	100	79.59
9	12,000	8	100	80.51
10	10,000	5	130	73.96
11	10,000	5	70	74.67
12	12,000	5	100	76.85
13	10,000	6.5	100	78.94
14	8000	5	100	70.54
15	10,000	6.5	100	83.61
16	10,000	6.5	100	80.79
17	10,000	8	70	72.66

**Table 4:** Response surface quadratic regression equation ANOVA results for high redox nanofly ash preparation process.

Source	Sum of Squares	*Df*	Mean Square	*F*-Value	*p*-Value	Significance
model	292.53	9	32.5	3.9	0.0433	significant
A	0.1081	1	0.1081	0.013	0.9126	-
B	41.68	1	41.68	5	0.0605	-
C	9.35	1	9.35	1.12	0.3249	-
AB	6.55	1	6.55	0.7855	0.4049	
AC	0.57	1	0.57	0.0683	0.8013	
BC	24.21	1	24.21	2.9	0.1323	
A^2^	78.51	1	78.51	9.41	0.0181	
B^2^	0.7401	1	0.7401	0.0887	0.7745	
C^2^	120.66	1	120.66	14.46	0.0067	
residual	58.4	7	8.34			
lost proposal	45.58	3	15.19	4.74	0.0834	not significant
pure error	12.82	4	3.2			
aggregate difference	350.93	16				

**Table 5:** Denitration efficiency of fly ash.

Temperature (°C)	Catalyst Type	NO Conversion Rate (%)
100	FA	71.435
200	FA	74.867
300	FA	76.691
400	FA	79.091
100	PFA	77.435
200	PFA	80.697
300	PFA	82.691
400	PFA	85.581

**Table 6:** Comparison of changes in compositional content of mixtures.

	SiO_2_	Fe_2_O_3_	Al_2_O_3_	CaO	K_2_O	TiO_2_
FA (%)	51.53	6.42	27.89	5.95	1.29	1.11
MSFA (%)	37.079	26.828	23.711	6.943	0.959	1.155
BMFA (%)	25.985	38.033	20.173	4.925	0.611	0.611
PFA (%)	27.789	37.461	22.379	6.582	0.69	1.088

**Table 7:** Comparison of changes in elemental content of monomers.

	O	Si	Fe	Al	Ca	K
FA (%)	65.286	15.368	0.742	11.702	3.277	0.855
MSFA (%)	42.915	17.322	18.764	12.549	4.962	0.797
BMFA (%)	38.689	12.679	28.384	11.085	3.705	0.533
PFA (%)	40.353	12.99	26.202	11.844	4.704	0.573

**Table 8:** Fly ash BET analysis data by stage.

Samples	Specific Surface Area (m^2^/g)	Total Pore Volume (cm^3^/g)	Average Pore Size (nm)
FA	2.3	0.0052	9.16
MSFA	3.2	0.0085	10.8
BMFA	14.5	0.0611	16.9
PFA	16.0	0.0786	19.6

**Table 9:** Pore size ANOVA results.

	Square Sum	*Df*	Mean Mquare	*F*	*p*
Constant	14.277	7	2.04	10.808	<0.01 **
B	11.551	2	5.776	18.278	<0.01 **
C	5.299	2	2.649	3.474	0.047 *
Pore size	1.698	9	0.189		

* *p* < 0.05, ** *p* < 0.01.

**Table 10:** Oxygroup ANOVA results.

	Square Sum	*Df*	Mean Square	*F*	*p*
Constant	91.820	7	13.117	3.792	0.034 *
B	59.03	2	29.51	16.4	0.252
C	84.272	2	42.136	15.25	<0.01 **
Oxygroup	31.135	9	3.459		

* *p* < 0.05, ** *p* < 0.01.

**Table 11:** Oxygroup and pore size variation results.

No.	Oxygroup (a.u.)	Average Pore Size (nm)	*OVI* (%)
1	48.5	20.7	80.59
2	55.9	20.7	69.36
3	54.5	17.9	72.82
4	54.8	18.9	68.49
5	47.8	20.8	81.79
6	50.9	20.2	79.32
7	50.7	18.9	73.46
8	53.1	20.6	79.59
9	52.7	19.6	80.51
10	51.8	19.1	73.96
11	48	19.3	74.67
12	49.6	19.8	76.85
13	49	20.1	78.94
14	55.6	18.4	70.54
15	47.9	21.5	83.61
16	49.8	20.7	80.79
17	52.7	18.9	72.66

**Table 12:** *OVI* linear regression analysis results.

	Normalization Coefficient	*T*	*P*	*VIF*	*R* ^2^	*F*
Constant		2.871	0.0944		0.692	*F* = 15.708, *p* < 0.05 *
Pore size	0.448	2.564	0.448	1.384		
Oxygroup	−0.504	−2.887	−0.504	1.384		

* *p* < 0.05, *DW* = 2.232.

## Data Availability

The original contributions presented in this study are included in the article. Further inquiries can be directed to the corresponding author.
